# Coinfection of Influenza A and B and Human OC43 Coronavirus in Normal Human Bronchial Epithelial Cells

**DOI:** 10.1111/irv.13279

**Published:** 2024-03-31

**Authors:** JungHyun Kim, Brady T. Hickerson, Natalia A. Ilyushina

**Affiliations:** ^1^ Division of Biotechnology Review and Research II Food and Drug Administration Silver Spring Maryland USA

**Keywords:** coinfection, cytokines, influenza, seasonal coronavirus

## Abstract

**Background:**

Influenza viruses and seasonal coronaviruses are pathogens transmitted via an airborne route that can cause respiratory diseases in humans that have similar symptoms such as fever, cough, and pneumonia. These two viruses can infect similar human tissues, such as the respiratory tract and nasal, bronchial, and alveolar epithelial cells. Influenza virus and seasonal coronavirus coinfections are poorly understood.

**Methods:**

Here, we coinfected normal human bronchial epithelial (NHBE) cells with influenza A/California/04/09 (IAV) or B/Victoria/504/2000 (IBV) strains and the seasonal human beta‐coronavirus OC43 and evaluated viral replication capacities. We also examined changes in the expression of various cytokines/chemokines by qPCR and Luminex assay.

**Results:**

We observed that the replication of IAV and IBV was not affected by coinfection with OC43. However, coinfection reduced OC43 titers (~3‐fold) compared with infection with OC43 alone. Select cytokine/chemokine expression was increased in coinfected cells compared with all single infections with greater differences seen between coinfected cells and cells infected with OC43 alone compared with IAV‐ or IBV‐infected cells. In addition, IL‐8 and IL‐1RA showed the highest expression among a panel of 22 cytokines by Luminex.

**Conclusions:**

As the rate of influenza and seasonal coronavirus coinfection continue to increase, our findings may help set guidelines for the treatments of the individuals coinfected with both viruses.

## 1 Introduction

Human coronaviruses and influenza A and B viruses (IAV and IBV, respectively) are of public health concern due to their potential to cause annual epidemics and pandemics with a huge economic burden for diagnosis and treatment. The severe acute respiratory syndrome coronavirus 2 (SARS‐CoV‐2) pandemic led to more than 765 million cases with about 7 million deaths worldwide (WHO). Additionally, the Centers for Disease Control and Prevention (CDC) [1] estimated that 140,000–710,000 people were hospitalized and 12,000–52,000 people died annually in the United States between 2010 and 2020 due to influenza virus infection (CDC). Moreover, novel influenza viruses emerge periodically causing global pandemics, with the most recent influenza pandemic occurred in 2009 [2].

Although public interests have recently been mainly focused on SARS‐CoV‐2, other endemic human betacoronaviruses circulate annually. In particular, OC43, HKU1, 229E, and NL63 are the four most common endemic coronavirus strains with OC43 being the most prevalent in terms of diagnosed cases [3]. Notably, seasonal coronaviruses and influenza viruses are transmitted via an airborne route and can infect the same human respiratory tract tissues including nasal and bronchial epithelial cells [4, 5]. Both virus infections can have similar symptoms like cough, fever, and pneumonia and are associated with lower respiratory tract diseases [6]. Additionally, seasonal endemic coronavirus infections overlap with influenza virus infections [7], and these viruses are expected to continue to co‐circulate in the human population, which presents the possibility of coinfection with these viruses.

Although seasonal coronavirus infection does not typically result in severe disease, coinfection with influenza virus has been associated with a more severe disease course [8, 9]. A previous epidemiological study demonstrated that coinfection of NL63 and IAV resulted in significantly higher rates of cough, sore throat, and difficulty of breathing in hospitalized patients compared with NL63 or IAV infection alone [9]. Moreover, clinical data showed that SARS‐CoV‐2 patients coinfected with IAV had higher incidences of ICU admission and higher mortality [10]. While Bai et al. [11] demonstrated that IAV preinfection significantly promoted SARS‐CoV‐2 infectivity both in vitro and in mice, preinfection with IAV suppressed SARS‐CoV‐2 replication in hamsters and did not affect IAV replication [12], demonstrating that results of coinfection may be dependent on the animal models used and the order of viral infection.

To our knowledge, there are no studies on seasonal coronavirus and influenza virus coinfection. Here, we coinfected normal human bronchial epithelial (NHBE) cells with OC43 and influenza A/California/04/09 (IAV) or B/Victoria/504/2000 (IBV) viruses and evaluated viral replication and cytokine/chemokine expression. Our findings provide a better understanding of the dynamics between OC43 and IAV or IBV coinfection in a human respiratory tract model.

## 2 Materials and Methods

### 2.1 Cells and Viruses

Human colorectal carcinoma cell line HCT‐8 (CCL‐244) cells were purchased from the American Type Culture Collection (ATCC, Manassas, VA, USA) and were cultured in DMEM with 10% fetal bovine serum (FBS). Madin–Darby canine kidney (MDCK) cells were purchased from ATCC and cultured in DMEM with 10% FBS. Primary human bronchial epithelial cells, NHBE, were obtained from Lonza (Walkersville, MD, USA) and cultured on membrane supports at the air‐liquid interface with serum‐free medium as described previously [13]. In all experiments, cells were fully differentiated for at least 3 weeks after being confluent.

OC43 (VR‐1558) was purchased from ATCC, and a virus stock was generated in HCT‐8 cells by passaging once for 6 days at 33°C in infection medium (DMEM with 2% FBS). Stocks of influenza A/California/04/09 (H1N1) and B/Victoria/504/2000 viruses were prepared by incubation of the viruses in the allantoic cavities of 10‐day‐old embryonated chicken eggs for 48 h at 37°C or 33°C for the IAV or IBV, respectively. All virus stocks were stored at −80°C. The experiments were performed in a BSL‐2 laboratory approved for use of OC43, H1N1, and IBV by the US Department of Agriculture and the US Center for Disease Control and Prevention.

### 2.2 Coinfection With Influenza (IAV or IBV) and OC43 Coronavirus

NHBE cells were washed three times with phosphate‐buffered saline (PBS) followed by infection with OC43 virus at multiplicity of infection (MOI) of 1 or IAV or IBV (MOI = 1). Cells were incubated for 2 h with OC43 virus at 33°C or for 1 h with IAV or IBV at 37°C or 33°C, respectively. Viruses were removed after incubation and were further incubated at the respective temperatures for 6 or 24 h. NHBE cells that were infected with influenza viruses were coinfected with OC43 (MOI = 1) and incubated for 2 h at 33°C. NHBE cells that were infected with OC43 virus, were coinfected with either IAV or IBV (MOI = 1) for 1 h. After the second infection, NHBE cells were incubated under air‐liquid interface conditions for 48 h at 33°C. NHBE cells used for single infection with the first virus were infected at the same time as the NHBE cells used for coinfection, indicating that the infection with the first virus lasted either 54 or 72 h in both single‐infected and coinfected cultures. NHBE cells used for single infection with the second virus were infected at the same time as the NHBE cells used for coinfection, indicating that the infection with the second virus lasted 48 h in both single‐infected and coinfected cultures. The supernatants were collected by the addition of 0.3 mL of media to the apical compartments, and aliquots were collected and stored at −80°C.

### 2.3 Influenza Infectivity Assay

The infectivity of the IAV and IBV viruses was determined by plaque assay [14]. Briefly, confluent cultures of MDCK cells were incubated for 1 h with 10‐fold serial dilutions of viruses at 37°C (IAV) or 33°C (IBV). The cells were then washed and overlaid with minimal essential medium containing 0.3% bovine serum albumin (BSA), 0.25% agarose, and 1 μg/mL l‐(tosylamido‐2‐phenyl) ethylchloromethylketone (TPCK)‐treated trypsin. After 3 days of incubation at 37°C (IAV) or 33°C (IBV), the cells were stained with 0.1% crystal violet in 10% formaldehyde solution, and the number of plaque‐forming units (PFU)/mL was determined. Values are the means of at least three independent determinations (with three replicates within each experiment).

### 2.4 OC43 Infectivity Assay

The infectivity of OC43 was determined by focus‐forming assay in HCT‐8 cells and expressed as focus‐forming units (FFU) per milliliter [15]. Briefly, confluent monolayers of HCT‐8 cells were incubated at 33°C for 2 h with 10‐fold serial dilutions of virus. The cells were then washed and overlaid with infection medium containing 1% carboxymethyl cellulose (CMC). After 5 days of incubation at 33°C, the cells were fixed with 10% formalin, permeabilized with 0.1% Triton X‐100, and blocked with PBS containing 1% BSA and 0.1% Tween‐20. OC43 antigen was stained with antibodies (primary: mouse anti‐OC43 nucleoprotein antibody [MAB9013; Millipore, Burlington, MA, USA], secondary: goat anti‐mouse horseradish peroxidase‐conjugated antibody [Sigma‐Aldrich, St. Louis, MO, USA]), visualized with SIGMAFast reagent (Sigma‐Aldrich), and FFU were visually quantified. Values are the means of at least three independent determinations (with three replicates within each experiment).

### 2.5 qPCR for Genes Expression

Quantification of changes in gene expression was carried out by quantitative real‐time PCR (qPCR) analyses of individual genes (*IFNB1*, *IFNL1*, *IFNL2/3*, *IFIT1*, *IFIT3*, *IFITM1*, *OAS1*, *MX1*, *IL‐6*, *CXCL10*, influenza matrix [*M1*], and OC3 nucleocapsid [*N*]). Total cellular RNA was harvested using the RNeasy minikit (Qiagen, Germantown, MD, USA). The collected RNA samples were treated with DNase, and then 1 μg of each purified RNA sample was reverse transcribed to cDNA with Quantiscript reverse transcriptase (Qiagen). The cDNAs were mixed with RT^2^ SYBR green qPCR Mastermix (Qiagen), and qPCR analyses were performed using the ViiA 7 instrument (Applied Biosystems, Waltham, MA). Influenza *M1* and OC43 *N* gene copy numbers were assayed using TaqMan gene expression assay primer/probe sets and master mix (Life Technologies, Carlsbad, CA, USA) and ViiA 7 software v.1.2.2 (Applied Biosystems). The values were determined by comparison to respective standard curves for each gene. Changes in gene expression levels were expressed as the mean‐fold increase relative to the untreated control gene expression levels after normalization to the housekeeping gene, *GAPDH*. Statistical analysis of the qPCR results was performed using Prism 9.0 (GraphPad Software, La Jolla, CA, USA). Values are the means of at least three independent determinations (with three replicates within each experiment).

### 2.6 Quantification of Cytokines/Chemokines by Luminex

Cytokine/chemokine levels in the supernatants of infected cells were measured using Milliplex Map human cytokine/chemokine multiplex assay kits (Millipore Sigma, Burlington, MA). Briefly, a 96‐well plate was washed with wash buffer and then diluted standards, controls, and samples were added to the plate together with antibody‐immobilized premixed beads. After an overnight incubation at 4°C with shaking, the plate was washed using a handheld magnet and then detection antibodies were added. After a 1 h incubation at room temperature on a plate shaker, streptavidin‐phycoerythrin was added to each well and the plate was incubated for 30 min at room temperature with shaking. The plate was then washed, 150 μL of Sheath fluid was added to all wells, and the cytokine/chemokine levels were measured by Luminex 200 and then analyzed by Belysa (Millipore Sigma). Cytokine/chemokine levels in the supernatants of the mock‐infected cells were also measured and were below the limit of detection (data not shown). Values are the means of at least two independent determinations (with three replicates within each experiment).

### 2.7 Statistical Analysis

Influenza and OC43 virus yields, levels of genes expression and cytokine/chemokine levels were compared by analysis of variance (ANOVA) using Prism 9.0 (GraphPad Software). Probability values ≤ 0.05 indicated statistically significant differences.

## 3 Results

### 3.1 Reduced OC43 Replication in NHBE Cells Coinfected With IAV or IBV

**FIGURE 1:**
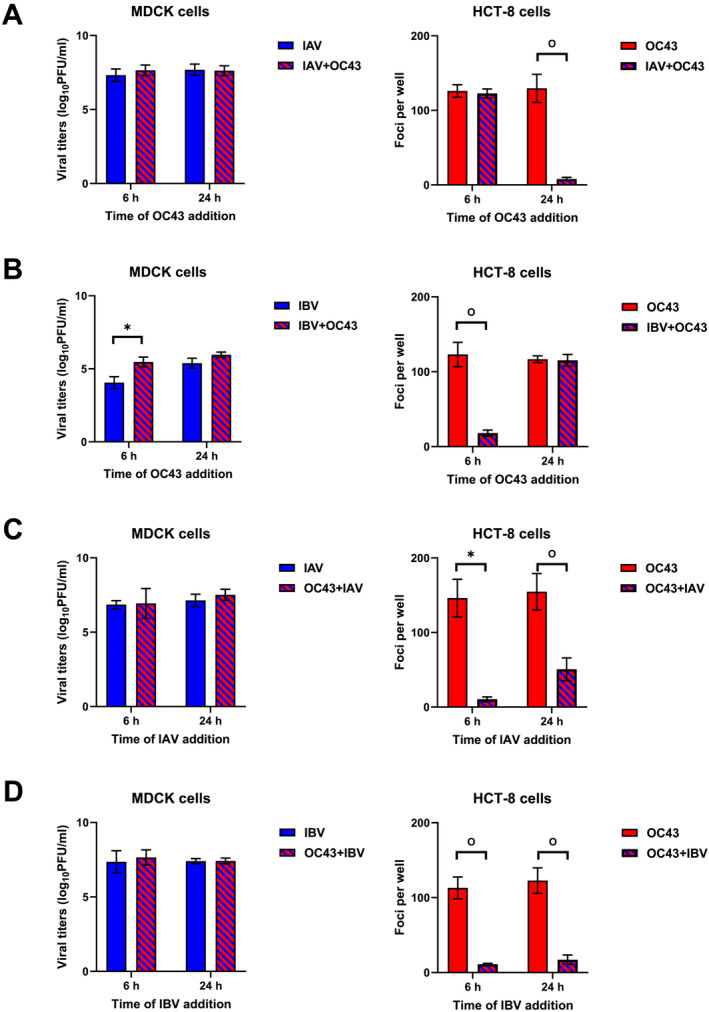
Infectivity of influenza and OC43 viruses. IAV viral titers quantified using MDCK cells and OC43 viral titers quantified using HCT‐8 cells in the following experimental conditions: (A) IAV infection followed by OC43 virus infection at 6 or 24 h (average from seven independent experiments is shown). (B) IBV infection followed by OC43 virus infection at 6 or 24 h (average from six independent experiments is shown). (C) OC43 virus infection followed by IAV infection at 6 or 24 h (average from three independent experiments is shown), and (D) OC43 virus infection followed by IBV infection at 6 or 24 h (average from three independent experiments is shown). Top of the bar is a mean of number of plaques or foci in duplicate or triplicate wells and error bars indicate a standard deviation. The figure shows the averaged results from three to seven independent experiments. FFU, focus‐forming units; PFU: plaque‐forming units.

To understand the effect of coinfection on infectivity and replication capacity of influenza and OC43 viruses in NHBE cells, we studied four different experimental conditions: infection with IAV or IBV followed by OC43 virus at 6 or 24 h after the first infection (Figure 1A,B) and infection with OC43 virus followed by IAV or IBV at 6 or 24 h after the first infection (Figure 1C,D). No changes in IAV and IBV viral titers and influenza *M* gene RNA copies were observed in the coinfected cells in all four experimental conditions (Figures 1 and S1). In contrast, OC43 virus yield was significantly reduced by 9‐fold when OC43 infection was followed by the IAV or 9‐fold when OC43 infection was followed by IBV compared with the OC43‐infected cells (Figure 1C,D). Moreover, OC43 virus yield was reduced by 16‐fold when IAV was followed by OC43 at 24 h or 7‐fold when IBV was followed by OC43 at 6 h after the first infection, compared with the cells infected with OC43 alone (Figure 1A,B). A similar trend was observed with OC43 *N* gene expression in NHBE lysates and supernatants (Figure S1). These data demonstrated that coinfection conditions did not affect IAV and IBV replication, but reduced OC43 infectivity.

### 3.2 Increased Levels of Cytokine/Chemokine Gene Expression in Coinfected Cells Compared With Single Influenza and OC43 Virus Infections

**FIGURE 2:**
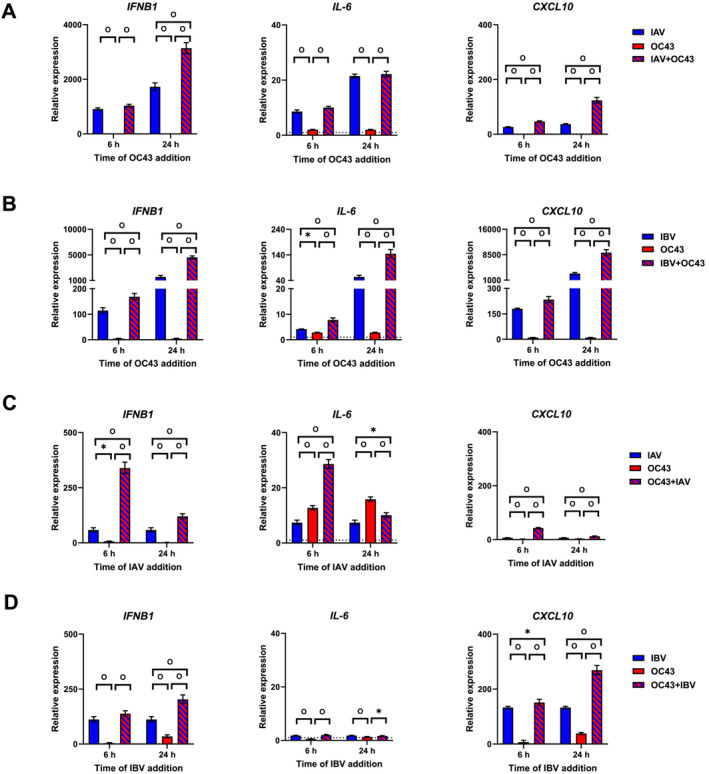
Quantification of cytokine/chemokine expressions by qPCR. Relative expression of *IFNB1*, *IL‐6*, and *CXCL10* genes as compared with uninfected control measured in NHBE cells after (A) IAV infection followed by OC43 virus infection at 6 or 24 h (average from seven independent experiments is shown). (B) IBV infection followed by OC43 virus infection at 6 or 24 h (average from six independent experiments is shown). (C) OC43 virus infection followed by IAV infection at 6 or 24 h (average from three independent experiments is shown), or (D) OC43 virus infection followed by IBV infection at 6 or 24 h (average from three independent experiments is shown). The dotted line indicates uninfected control. Top of the bar indicates a mean of duplicate samples with error bars for standard deviations. The figure shows the averaged results from three to seven independent experiments. *p < 0.05 and °*p* < 0.01.

We next assessed expression levels of various cytokines and chemokines (i.e., *IFNB1*, *IFNL1*, *IFNL2/3*, *IFIT1*, *IFIT3*, *OAS1*, *MX1*, *IL‐6*, *CXCL10*, and *IFITM1*) which were triggered by influenza and OC43 virus infections alone or together by qPCR. When IAV or IBV infection was followed by OC43, cytokine/chemokine levels were significantly higher in IAV‐ (57‐fold) or IBV‐infected (341‐fold) cells than in those infected with OC43 (Figure 2A,B). We observed that most of the genes (i.e., *IFNB1*, *IFNL1*, *IFNL2/3*, *IFIT1*, *IFIT3*, *OAS1*, and *CXCL10*) were expressed significantly higher in NHBE cells coinfected with both viruses when OC43 virus infection followed IAV 24 h postinfection compared with IAV (2‐fold) or OC43 virus (219‐fold) infection alone (Figures 2A and S2). Moreover, the expression levels of *IFNB1*, *IFNL1*, *IFNL2/3*, *IL‐6*, and *CXCL10* were significantly higher in the coinfected cultures as compared with those infected with IBV (2‐fold) or OC43 (from 3‐ to 12,719‐fold) alone independent on the time of OC43 addition (Figures 2B and S3).

Similar patterns were observed when OC43 virus infection was followed either by IAV or IBV:cytokine/chemokine levels were higher in influenza virus‐infected cells (5‐fold for IAV and 7‐fold for IBV) than in those infected with OC43 alone (Figures 2C,D, S4, and S5). Coinfection induced significantly higher levels of all studied genes except *IL‐6* compared with IAV (3‐fold) or OC43 (16‐fold) single infections (Figures 2C and S4). We also observed that most of the genes (i.e., *IFNL1*, *IFNL2/3*, *IFIT1*, *IFIT3*, *OAS1*, *MX1*, *IFITM1*, and *CXCL10*) were expressed to higher levels in coinfected cultures compared with IBV (2‐fold) or OC43 virus (11‐fold) alone when OC43 was followed by IBV 6 or 24 h postinfection (Figures 2D and S5). Taken together, coinfection with influenza and OC43 induced significantly higher levels of the cytokine/chemokine gene expression compared with single infections, with the most significant differences seen between coinfection and OC43 virus infection.

We also compared cytokine/chemokine expression levels of the coinfected samples between two time points (i.e., 6 h vs. 24 h after addition of the first virus). The 24 h samples demonstrated higher expression levels of all genes compared with 6 h samples: 3‐fold or 30‐fold when IAV or IBV was followed by OC43 virus, respectively (Figures 2A,B, S2, and S3). In contrast, expression levels decreased or did not change in 24 h coinfected cultures compared with 6 h samples when OC43 infection was followed by IAV or IBV (Figures 2C,D, S4, and S5). Our results demonstrated that OC43 infection was able to boost inflammatory response in cells initially infected with IAV or IBV whereas IAV or IBV did not affect cytokine/chemokine expression in OC43 virus‐infected cells.

### 3.3 High Expression of IL‐1RA and IL‐8 in Cultures Infected With Influenza and OC43 Viruses Used Alone and Together

**FIGURE 3:**
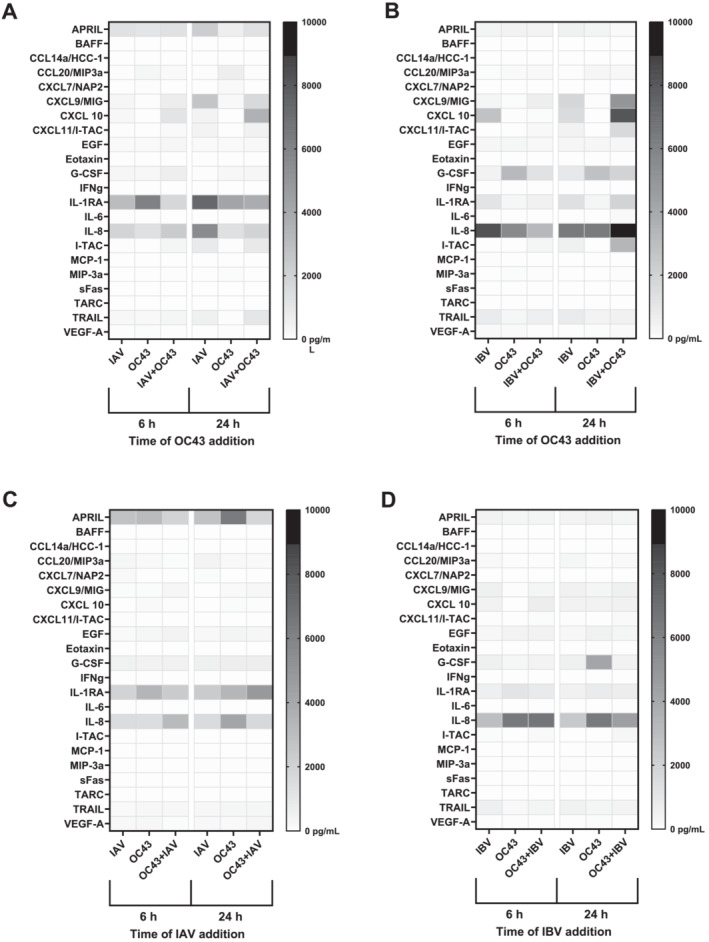
Heatmap of cytokine/chemokine expression measured by Luminex. Cytokine/chemokine expression in the supernatants collected from NHBE cells infected with (A) IAV followed by OC43 virus at 6 or 24 h, (B) IBV followed by OC43 virus at 6 or 24 h, (C) OC43 virus followed by IAV at 6 or 24 h, or (D) OC43 virus followed by IBV at 6 or 24 h. Expression of all measured proteins was in pg/mL range and darker color indicates higher expression. The figure shows the averaged results from two independent experiments.

We next measured protein expression of 22 cytokines and chemokines by Luminex. We observed that IL‐1RA, an anti‐inflammatory cytokine, and IL‐8, a pro‐inflammatory chemokine, were highly expressed in all four experimental conditions compared with the rest of the inflammatory proteins studied (Figure 3). Interestingly, IL‐1RA expression was 3‐fold higher in cultures coinfected with IAV and OC43 as compared with those coinfected with IBV and OC43 virus (Figures 3, S6, and S8). In contrast, IL‐8 expression was 3‐fold higher in cells coinfected with IBV and OC43 compared with coinfection with IAV and OC43 (Figures 3, S7, and S9). Additionally, a proliferating‐inducing ligand (APRIL) was highly expressed only in NHBE cultures infected with IAV and OC43 (Figure 3A,C) and granulocyte‐colony stimulating factor (G‐CSF), which promote pro‐inflammatory response in lungs during viral infection, was moderately expressed in cells infected with IBV and OC43 (Figure 3B,D). Two pro‐inflammatory cytokines, chemokine ligand 9 (CXCL9) and 10 (CXCL10), were moderately expressed in cultures infected with IAV or IBV followed by OC43 virus (Figure 3A,B). In addition, our results showed that expression levels of IL‐6 and CXCL10 measured by Luminex corroborated well with the respective gene expression levels measured by qPCR. In particular, IL‐6 expression in cultures coinfected with IAV followed by OC43 24 h postinfection was higher compared with IAV‐ and OC43‐infected cells (3‐fold and 5‐fold, respectively, Figures 3A and S6). IBV followed by OC43 24 h postinfection induced higher expression of IL‐6 compared with IAV (9‐fold) and OC43 (83‐fold) single infections (Figures 3B and S7). CXCL10 protein expression was higher in cultures infected with IAV or IBV followed by OC43 24 h postinfection compared with influenza‐ (7‐fold) or OC‐43‐infected (162‐fold) cells (Figures 3A,B, S6, and S7). In addition, OC43 infection followed by IBV 6 h postinfection triggered higher expression of CXCL10 in coinfected cultures compared with IBV and OC43 (2‐fold and 21‐fold, respectively, Figures 3D and S9).

## 4 Discussion

As the seasonal coronavirus and influenza viruses' coinfection frequency continues to rise, it is important to understand the interaction between both viruses within a coinfected host. In this study, NHBE cells were coinfected with seasonal OC43 coronavirus and IAV or IBV viruses and replication capacity and cytokine/chemokine expressions were examined in comparison with single virus infections. We observed that coinfection did not affect infectivity of IAV and IBV, whereas OC43 virus infectivity was significantly reduced. A similar pattern was seen when influenza *M gene* and OC43 viral *N gene* RNA copies were measured by qPCR. Coinfection also induced expression of numerous cytokines/chemokines compared with single virus infections with greater difference observed for OC43‐infected cells versus influenza single infections.

To the best of our knowledge, our study is the first report examining seasonal coronavirus and influenza virus coinfection. Our findings correlated well with a previous study by Oishi et al. [12] demonstrating that hamsters infected with IAV followed by SARS‐CoV‐2 suppressed SARS‐CoV‐2 replication whereas preinfection with SARS‐CoV‐2 followed by IAV did not affect IAV replication. In contrast, while Oishi group demonstrated that hamsters preinfected with SARS‐CoV‐2 followed by IAV did not affect replication of both IAV and SARS‐CoV‐2, we observed that preinfection with OC43 virus followed by IAV or IBV significantly reduced OC43 virus titers in coinfected NHBE cultures. Moreover, coinfection of SARS‐CoV‐2 and IAV in a mouse model showed increased SARS‐CoV‐2 viral load [11] and coinfection reduced influenza virus replication in A549‐ACE2 cells but not in Vero‐E6 cells [12]. This emphasizes that different results can be observed depending on which in vitro or in vivo models are used and there are multiple unknown factors besides types of viruses that can impact virus replication capacity within a coinfected host.

We observed that relatively low levels of cytokines/chemokines were expressed in OC43‐infected cultures, indicating that infection with this virus was not capable to induce substantial inflammatory response in the epithelial cells. These results correlate well with the previously published study where OC43 virus was shown to trigger low or absolutely no expression of type I and III interferons and interferon‐stimulated genes (ISGs) compared with alphacoronavirus 229E in primary human bronchial epithelial cells [16]. Moreover, COVID‐19 patients demonstrated a small number of elevated cytokines compared with influenza‐infected patients [17]. This explains, at least partially, why IAV and IBV promoted higher inflammation compared with OC43 virus infection in the present study. In addition, higher IFNs and ISGs expression triggered by IAV and IBV as compared with OC43 can explain, at least partially, reduced OC43 replication in the coinfected NHBE cells in all four experimental conditions. Indeed, Oishi group demonstrated that *IRF7* and *ISG15* genes expression remained elevated in the IAV‐recovered hamsters, and this reduced SARS‐CoV‐2 replication after IAV clearance [12]. Moreover, whereas ISGs expression peaked on day 3 in the hamsters' airways after infection with IAV, the ISGs reached the highest level on days 5–7 postinfection with SARS‐CoV‐2 [18]. This suggests that inflammatory response triggered by one virus can interfere with that triggered by the other virus in the co‐infected host and it can also affect replication activities of both viruses. Overall, more studies are needed to investigate the mechanism of reduction of OC43 replication in the coinfected epithelial cells.

IL‐8 is a chemotactic protein that plays an important role in inflammation and its expression is increased during viral infections in vivo and in vitro [19, 20]. In contrast, IL‐1RA is an anti‐inflammatory protein that occurs naturally to inhibit proinflammatory activity induced by IL‐1 [21]. During influenza virus infection, human airway epithelial cells actively induce IL‐8 [22, 23]. Additionally, influenza‐infected patients showed increased level of IL‐1RA and treatment with IL‐1RA increased survival rate of mice infected with influenza virus A/PR/8/34 [24, 25]. IL‐8 and IL‐1RA are good biomarkers for COVID‐19 disease severity. IL‐8 levels were distinctively different in patients at severe or recovered stages and IL‐1RA expression was significantly increased during the early stage of severe COVID‐19 infection in humans [26, 27]. In a good alignment with these results, we observed elevated expression of IL‐8 and IL‐1RA in both single and coinfected samples in all four experimental conditions by Luminex assay. Further studies are needed to understand why coinfection of IAV and OC43 promoted the expression of the anti‐inflammatory cytokine IL‐1RA, whereas IBV and OC43 triggered expression of the pro‐inflammatory cytokine IL‐8.

As SARS‐CoV‐2 infections decrease, seasonal coronavirus infections will likely increase. To the best of our knowledge, our study is the first on influenza virus and seasonal coronavirus coinfection in human epithelial cells. Our findings shed a light on the interaction between the two types of viruses and may help to further guide treatment control strategies for these human pathogens.

## Author Contributions

**JungHyun Kim:** Formal analysis; Investigation; Writing – original draft. **Brady T. Hickerson:** Data curation; Methodology; Writing – review and editing. **Natalia A. Ilyushina:** Conceptualization; Funding acquisition; Project administration; Supervision; Writing – original draft; Writing – review and editing.

## Disclosure

The article reflects the views of the authors and should not be construed to represent FDA's views or policies.

## Conflicts of Interest

The authors declare no conflicts of interest.

### Peer Review

The peer review history for this article is available at https://www.webofscience.com/api/gateway/wos/peer‐review/10.1111/irv.13279.

## Supporting information


**Figure S1.** Quantification of influenza virus *M gene* or OC43 viral *N gene* by qPCR. *M or N gene* copy numbers were quantified by qPCR with RNA isolated from supernatant or lysates of infected NHBE cells in the following experimental conditions: (A) IAV infection followed by OC43 virus infection at 6 or 24 hours (average from seven independent experiments is shown), (B) IBV infection followed by OC43 virus infection at 6 or 24 hours (average from six independent experiments is shown), (C) OC43 virus infection followed by IAV infection at 6 or 24 hours (average from three independent experiments is shown), and (D) OC43 virus infection followed by IBV infection at 6 or 24 hours (average from three independent experiments is shown). Top of the bar graph is a mean of triplicate wells and error bars indicate standard deviations. The figure shows the averaged results from three to seven independent experiments.
**Figure S2.** Cytokine/chemokine expression in NHBE cells infected with IAV followed by OC43 virus. Relative expression of cytokines/chemokines in NHBE cells infected with IAV alone, OC43 virus alone, or IAV infected 6 hr or 24 hr prior to coinfection with OC43 virus. Expressions were compared with uninfected control cells. Top of the bar indicates a mean of duplicate or triplicate wells and error bars represent standard deviations. The dotted line indicates uninfected control. The figure shows the averaged results from three to seven independent experiments. * *p* < 0.05 and ° *p* < 0.01.
**Figure S3.** Cytokine/chemokine expression in NHBE cells infected with IBV followed by OC43 virus. Relative expression of cytokines/chemokines in NHBE cells infected with IBV alone, OC43 virus alone, or IBV infected 6 hr or 24 hr prior to coinfection with OC43 virus. Expressions were compared with uninfected control cells. Top of the bar indicates a mean of duplicate or triplicate wells and error bars represent standard deviations. The dotted line indicates uninfected control. The figure shows the averaged results from three to seven independent experiments. * *p* < 0.05 and ° *p* < 0.01.
**Figure S4.** Cytokine/chemokine expression in NHBE cells infected with OC43 virus followed by IAV. Relative expression of cytokines/chemokines in NHBE cells infected with OC43 virus alone, IAV alone, or OC43 virus infected 6 hr or 24 hr prior to coinfection with IAV. Expressions were compared with uninfected control cells. Top of the bar indicates a mean of duplicate or triplicate wells and error bars represent standard deviations. The dotted line indicates uninfected control. The figure shows the averaged results from three to seven independent experiments. * *p* < 0.05 and ° *p* < 0.01.
**Figure S5.** Cytokine/chemokine expression in NHBE cells infected with OC43 virus followed by IBV. Relative expression of cytokines/chemokines in NHBE cells infected with OC43 virus alone, IBV alone, or OC43 virus infected 6 hr or 24 hr prior to coinfection with IBV. Expressions were compared with uninfected control cells. Top of the bar indicates a mean of duplicate or triplicate wells and error bars represent standard deviations. The dotted line indicates uninfected control. The figure shows the averaged results from three to seven independent experiments. * *p* < 0.05 and ° *p* < 0.01.
**Figure S6.** Quantification of cytokines/chemokines by Luminex in NHBE cells infected with IAV followed by OC43 virus. Cytokine/chemokine expression were measured from supernatant taken from infected NHBE cells using Luminex panels. Top of the bar indicates a mean of duplicate or triplicate wells and error bars represent standard deviations. The figure shows the averaged results from two independent experiments. * *p* < 0.05 and ° *p* < 0.01.
**Figure S7.** Quantification of cytokines/chemokines by Luminex in NHBE cells infected with IBV followed by OC43 virus. Cytokine/chemokine expression were measured from supernatant taken from infected NHBE cells using Luminex panels. Top of the bar indicates a mean of duplicate or triplicate wells and error bars represent standard deviations. The figure shows the averaged results from two independent experiments. * *p* < 0.05 and ° *p* < 0.01.
**Figure S8.** Quantification of cytokines/chemokines by Luminex in NHBE cells infected with OC43 virus followed by IAV. Cytokine/chemokine expression were measured from supernatant taken from infected NHBE cells using Luminex panels. Top of the bar indicates a mean of duplicate or triplicate wells and error bars represent standard deviations. The figure shows the averaged results from two independent experiments. * *p* < 0.05 and ° *p* < 0.01.
**Figure S9.** Quantification of cytokines/chemokines by Luminex in NHBE cells infected with OC43 virus followed by IBV. Cytokine/chemokine expression were measured from supernatant collected from infected NHBE cells using Luminex panels. Top of the bar indicates a mean of duplicate or triplicate wells and error bars represent standard deviations. The figure shows the averaged results from two independent experiments. * *p* < 0.05 and ° *p* < 0.01.

## Data Availability

The data that support the finding of this study are available from the corresponding author upon reasonable request.
