# Supplementary material for: Coinfection of Influenza A and B and Human OC43 Coronavirus in Normal Human Bronchial Epithelial Cells
Source: Influenza Other Respir Viruses. 2024 Mar 31;18(4):e13279. doi: 10.1111/irv.13279 (PMC10982074; doi:10.1111/irv.13279)
Supplement: Supplementary file 1 — Figure S1. Quantification of influenza virus M gene or OC43 viral N gene by qPCR. M or N gene copy numbers were quantified by qPCR with RNA isolated from supernatant or lysates of infected NHBE cells in the following experimental conditions: (A) IAV infection followed by OC43 virus infection at 6 or 24 hours (average from seven independent experiments is shown), (B) IBV infection followed by OC43 virus infection at 6 or 24 hours (average from six independent experiments is shown), (C) OC43 virus infection followed by IAV infection at 6 or 24 hours (average from three independent experiments is shown), and (D) OC43 virus infection followed by IBV infection at 6 or 24 hours (average from three independent experiments is shown). Top of the bar graph is a mean of triplicate wells and error bars indicate standard deviations. The figure shows the averaged results from three to seven independent experiments. Figure S2. Cytokine/chemokine expression in NHBE cells infected with IAV followed by OC43 virus. Relative expression of cytokines/chemokines in NHBE cells infected with IAV alone, OC43 virus alone, or IAV infected 6 hr or 24 hr prior to coinfection with OC43 virus. Expressions were compared with uninfected control cells. Top of the bar indicates a mean of duplicate or triplicate wells and error bars represent standard deviations. The dotted line indicates uninfected control. The figure shows the averaged results from three to seven independent experiments. * p < 0.05 and ° p < 0.01. Figure S3. Cytokine/chemokine expression in NHBE cells infected with IBV followed by OC43 virus. Relative expression of cytokines/chemokines in NHBE cells infected with IBV alone, OC43 virus alone, or IBV infected 6 hr or 24 hr prior to coinfection with OC43 virus. Expressions were compared with uninfected control cells. Top of the bar indicates a mean of duplicate or triplicate wells and error bars represent standard deviations. The dotted line indicates uninfected control. The figure show [file IRV-18-e13279-s001.pdf]

Figure S1

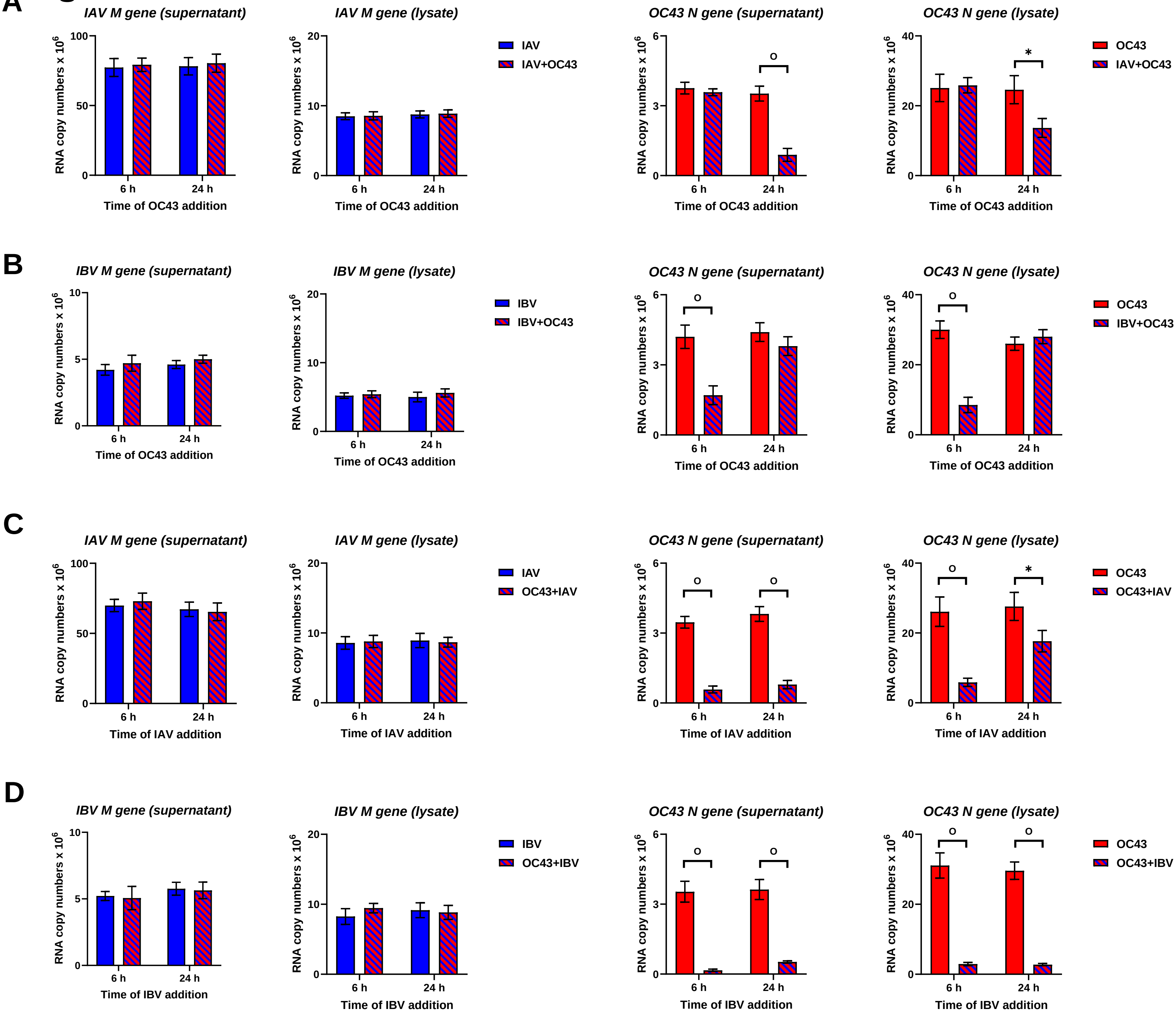
A
IAV M gene (supernatant)
IAV M gene (lysate)
IAV
IAV+OC43
OC43 N gene (supernatant)
OC43 N gene (lysate)
OC43
IAV+OC43
RNA copy numbers x 10^6
6 h
24 h
Time of OC43 addition
B
IBV M gene (supernatant)
IBV M gene (lysate)
IBV
IBV+OC43
OC43 N gene (supernatant)
OC43 N gene (lysate)
OC43
IBV+OC43
Time of OC43 addition
C
IAV M gene (supernatant)
IAV M gene (lysate)
IAV
OC43+IAV
OC43 N gene (supernatant)
OC43 N gene (lysate)
OC43
OC43+IAV
Time of IAV addition
D
IBV M gene (supernatant)
IBV M gene (lysate)
IBV
OC43+IBV
OC43 N gene (supernatant)
OC43 N gene (lysate)
OC43
OC43+IBV
Time of IBV addition

Figure S2

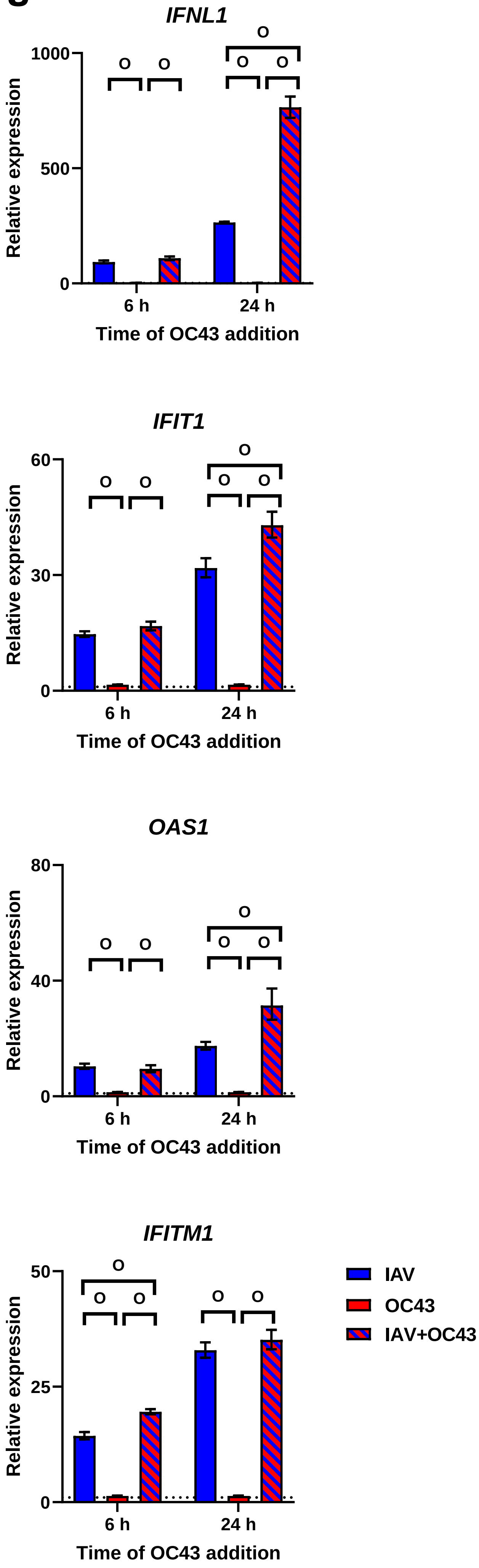
IFNL1
IFNL2/3
IFIT1
IFIT3
OAS1
MX1
IFITM1
Relative expression
Time of OC43 addition
6 h
24 h
IAV
OC43
IAV+OC43

Figure S3

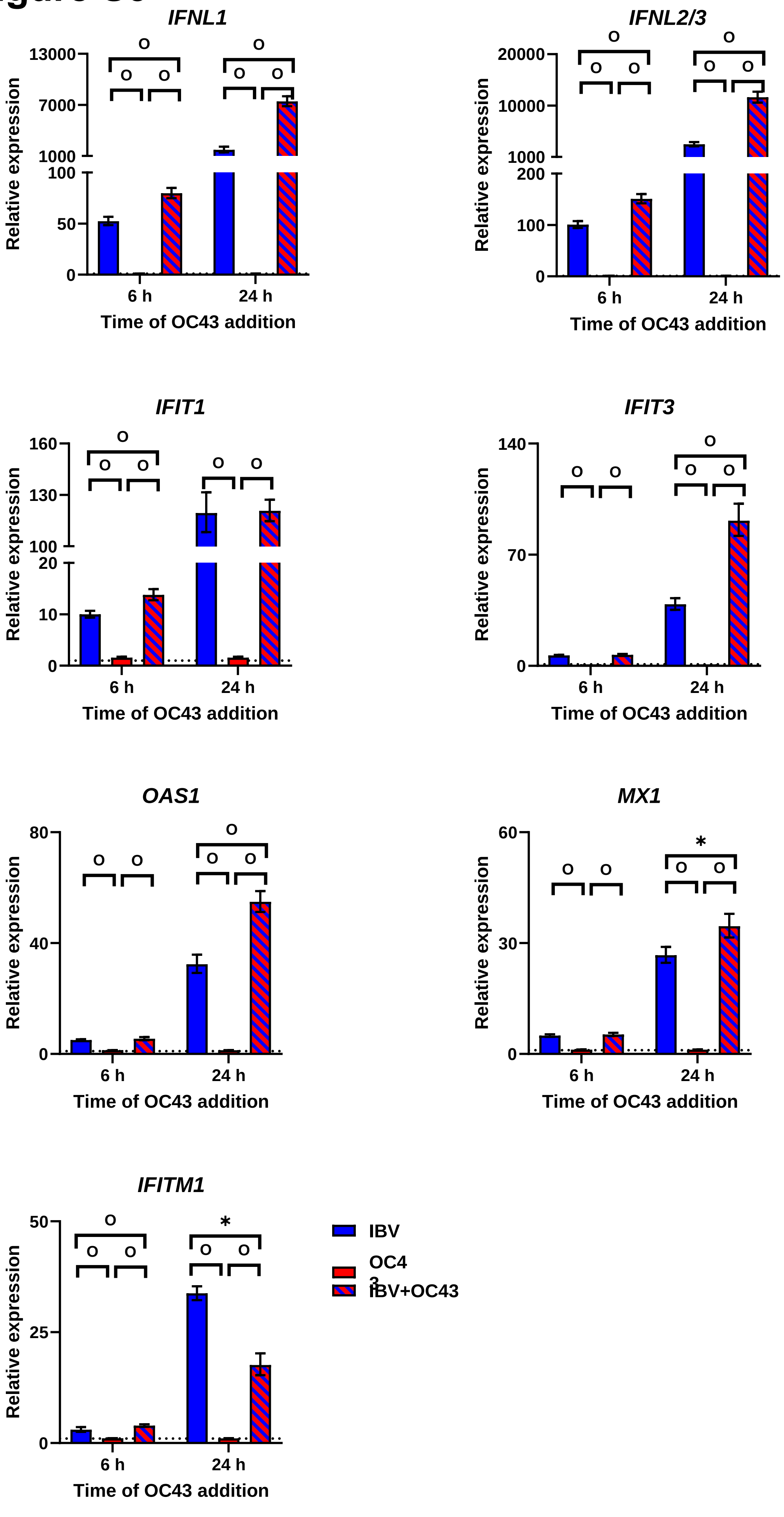
IFNL1
IFNL2/3
IFIT1
IFIT3
OAS1
MX1
IFITM1
Relative expression
Time of OC43 addition
6 h
24 h
IBV
OC4
3
IBV+OC43

Figure S4

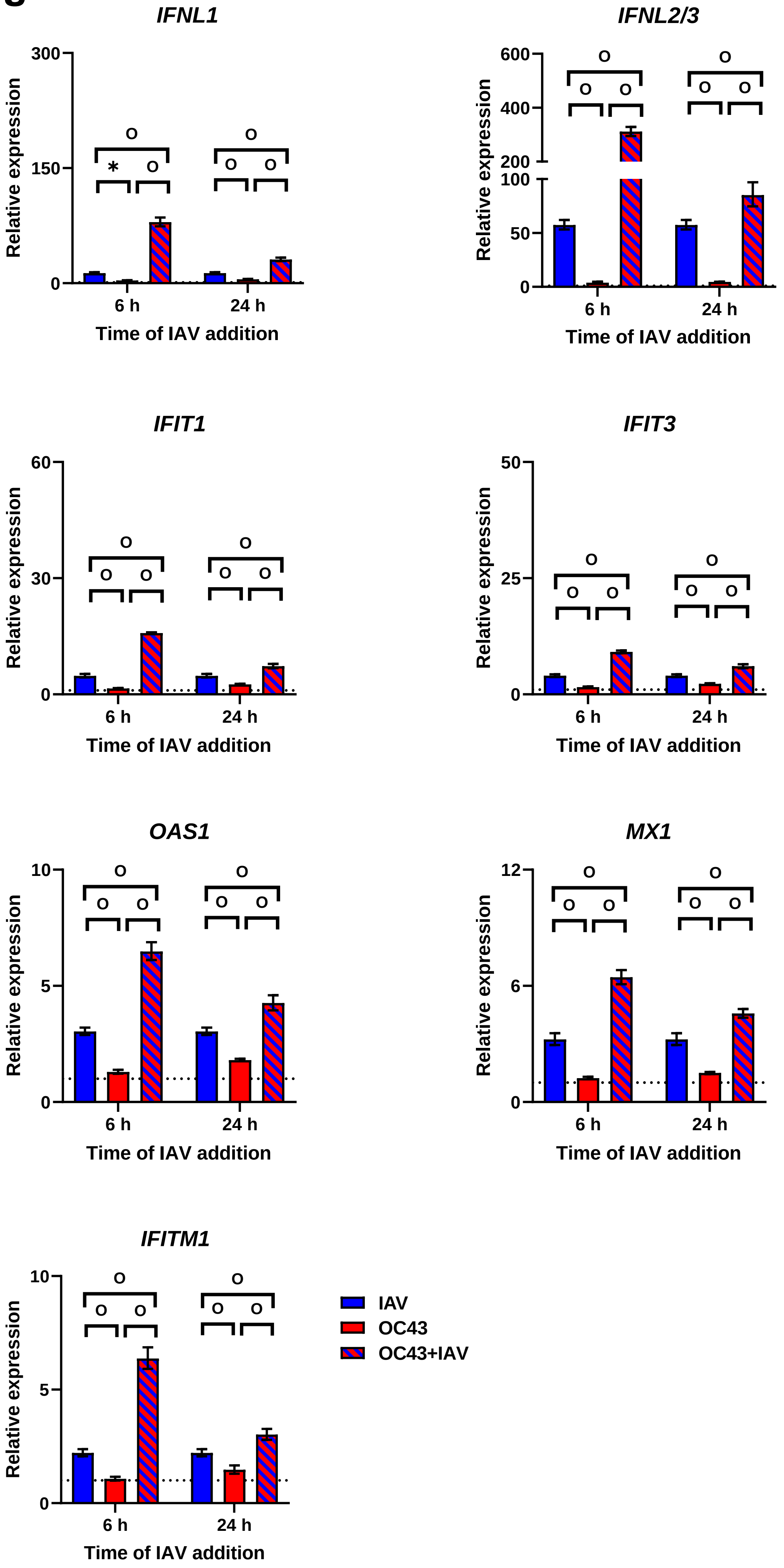
IFNL1
IFNL2/3
IFIT1
IFIT3
OAS1
MX1
IFITM1
Relative expression
6 h
24 h
Time of IAV addition
IAV
OC43
OC43+IAV

Figure S5

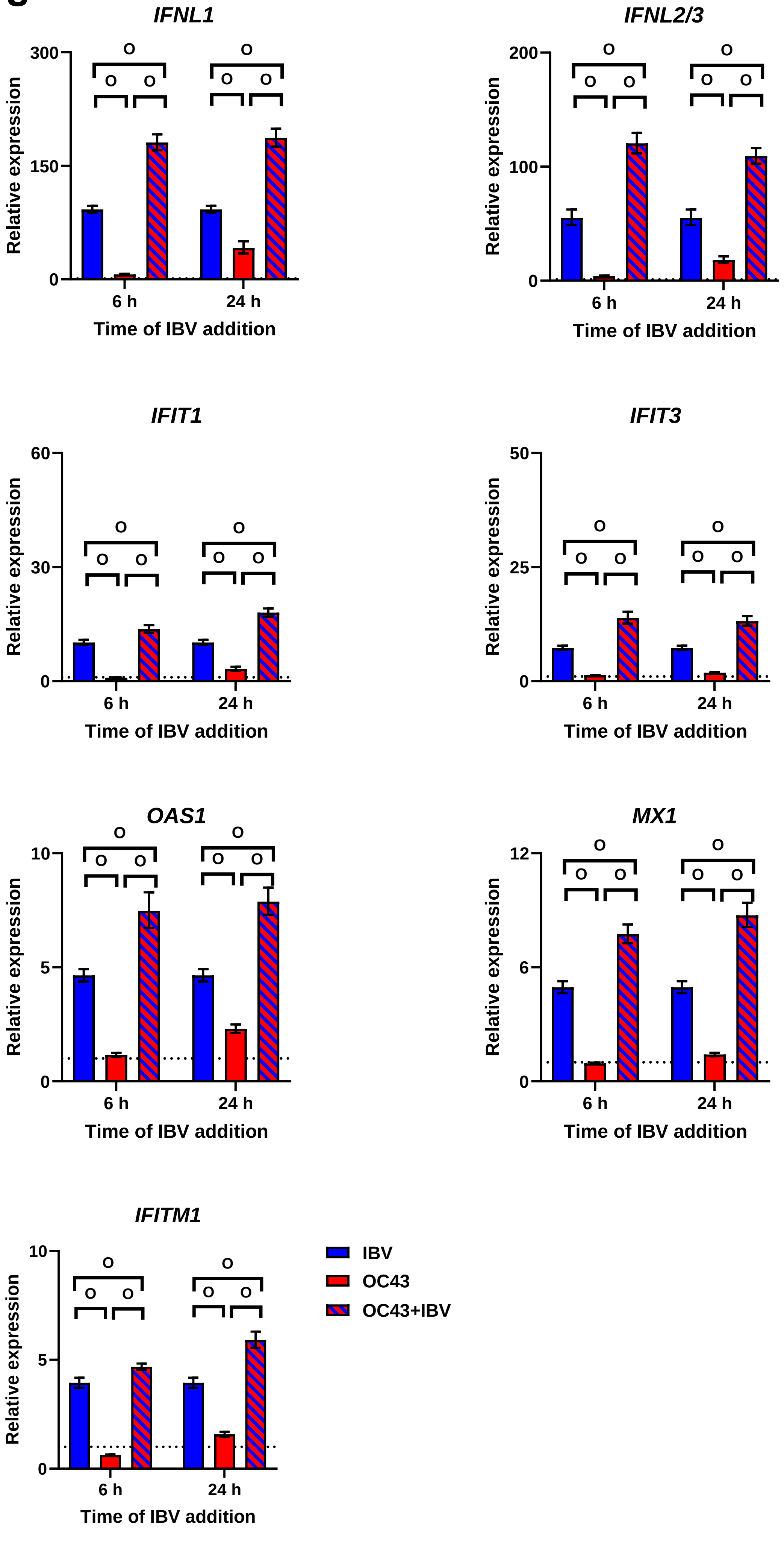
IFNL1
IFNL2/3
IFIT1
IFIT3
OAS1
MX1
IFITM1
Relative expression
Time of IBV addition
6 h
24 h
IBV
OC43
OC43+IBV

Figure S6

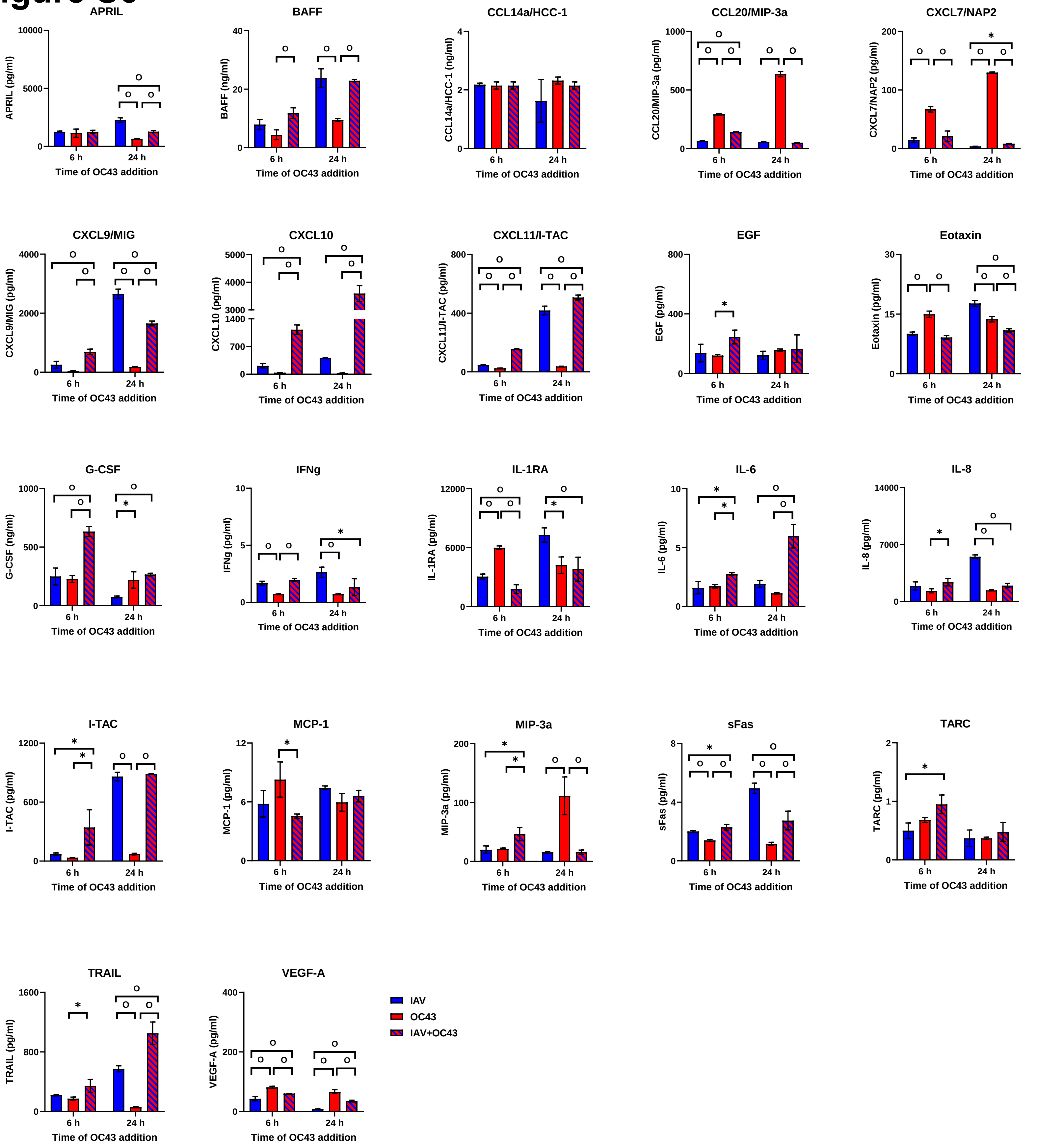

APRIL
BAFF
CCL14a/HCC-1
CCL20/MIP-3a
CXCL7/NAP2
CXCL9/MIG
CXCL10
CXCL11/I-TAC
EGF
Eotaxin
G-CSF
IFNg
IL-1RA
IL-6
IL-8
I-TAC
MCP-1
MIP-3a
sFas
TARC
TRAIL
VEGF-A
Time of OC43 addition
6 h
24 h
IAV
OC43
IAV+OC43

Figure S7

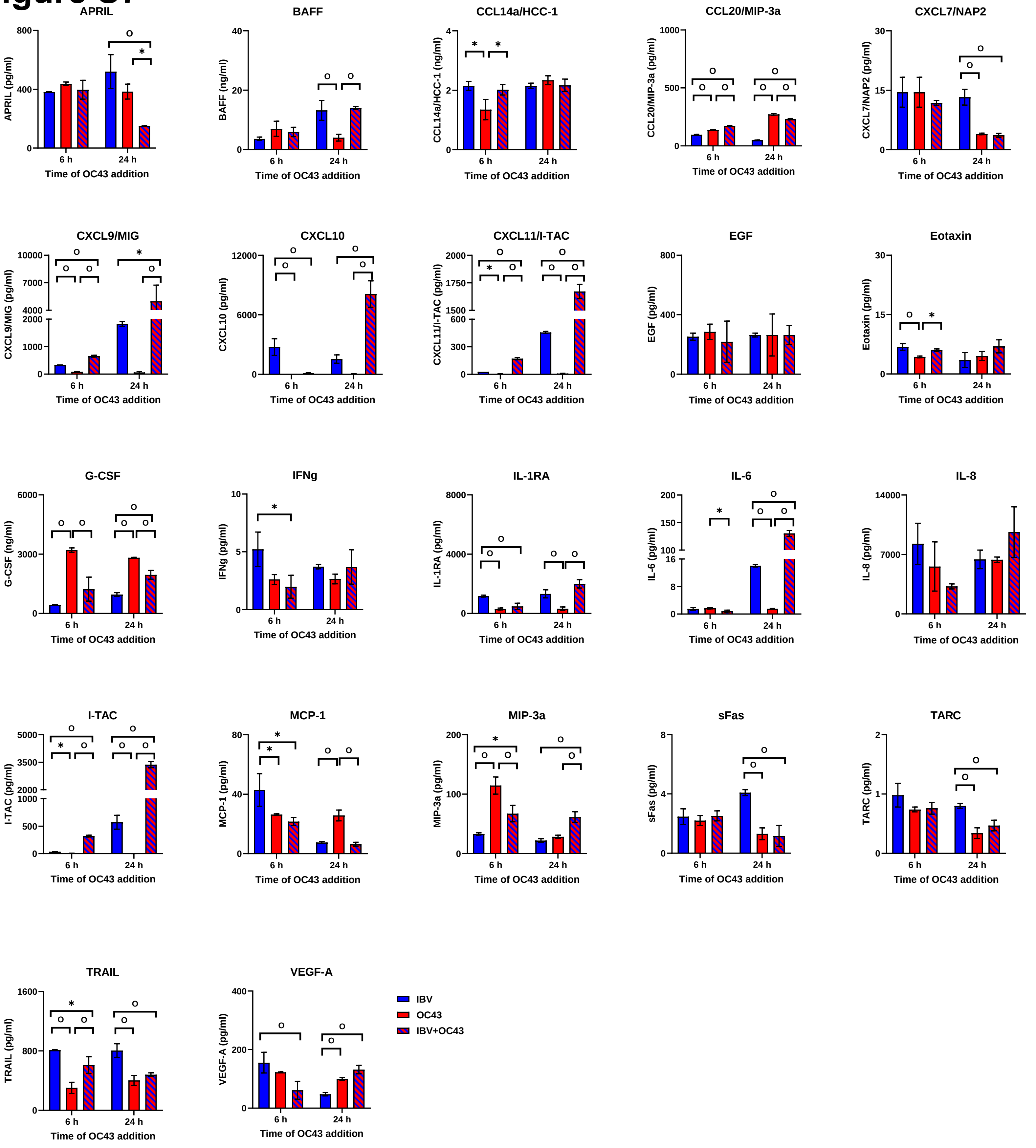
APRIL
APRIL (pg/ml)
BAFF
BAFF (ng/ml)
CCL14a/HCC-1
CCL14a/HCC-1 (ng/ml)
CCL20/MIP-3a
CCL20/MIP-3a (pg/ml)
CXCL7/NAP2
CXCL7/NAP2 (pg/ml)
CXCL9/MIG
CXCL9/MIG (pg/ml)
CXCL10
CXCL10 (pg/ml)
CXCL11/I-TAC
CXCL11/I-TAC (pg/ml)
EGF
EGF (pg/ml)
Eotaxin
Eotaxin (pg/ml)
G-CSF
G-CSF (ng/ml)
IFNg
IFNg (pg/ml)
IL-1RA
IL-1RA (pg/ml)
IL-6
IL-6 (pg/ml)
IL-8
IL-8 (pg/ml)
I-TAC
I-TAC (pg/ml)
MCP-1
MCP-1 (pg/ml)
MIP-3a
MIP-3a (pg/ml)
sFas
sFas (pg/ml)
TARC
TARC (pg/ml)
TRAIL
TRAIL (pg/ml)
VEGF-A
VEGF-A (pg/ml)
6 h
24 h
Time of OC43 addition
IBV
OC43
IBV+OC43

Figure S8

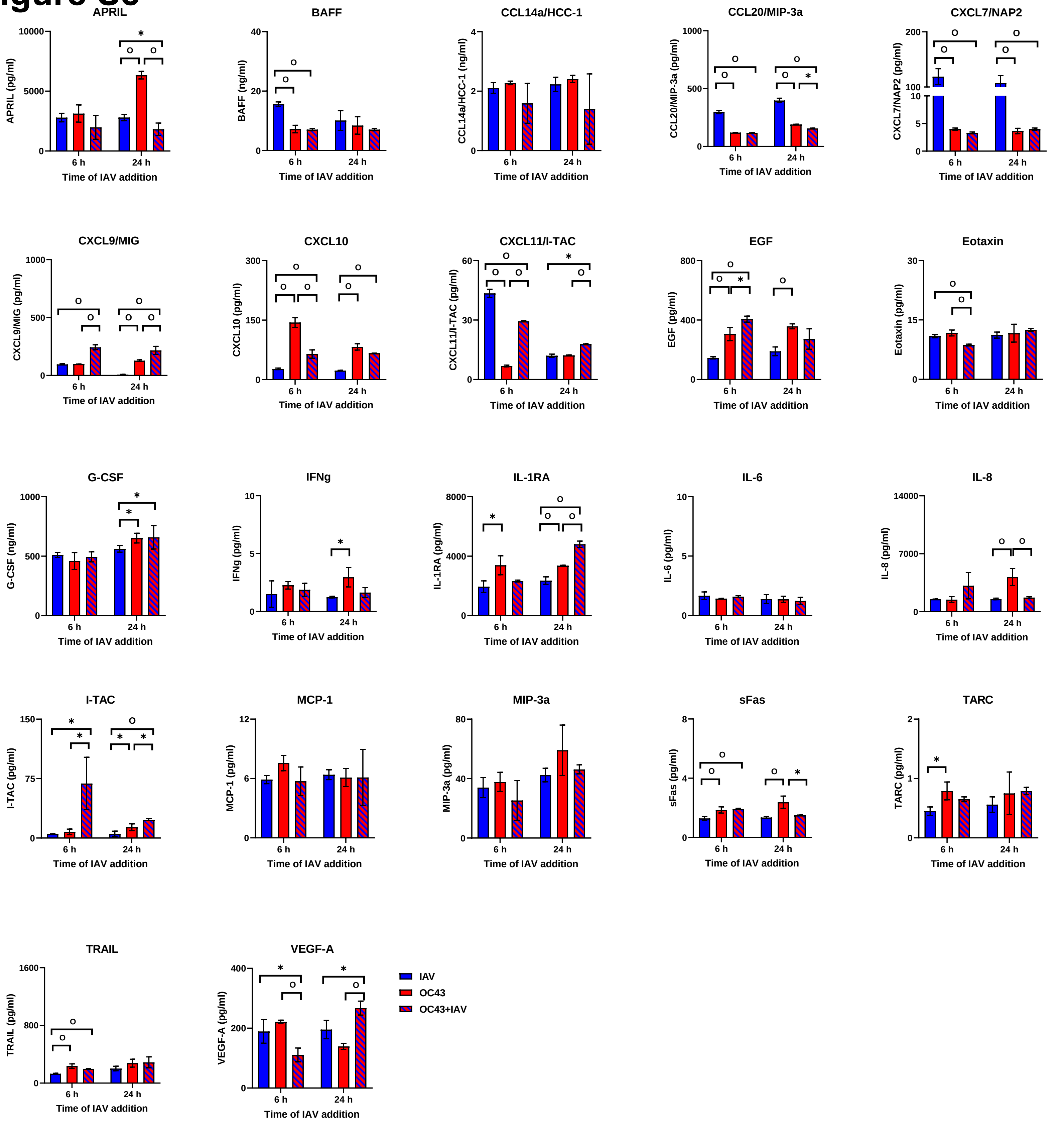

APRIL
APRIL (pg/ml)
BAFF
BAFF (ng/ml)
CCL14a/HCC-1
CCL14a/HCC-1 (ng/ml)
CCL20/MIP-3a
CCL20/MIP-3a (pg/ml)
CXCL7/NAP2
CXCL7/NAP2 (pg/ml)
CXCL9/MIG
CXCL9/MIG (pg/ml)
CXCL10
CXCL10 (pg/ml)
CXCL11/I-TAC
CXCL11/I-TAC (pg/ml)
EGF
EGF (pg/ml)
Eotaxin
Eotaxin (pg/ml)
G-CSF
G-CSF (ng/ml)
IFNg
IFNg (pg/ml)
IL-1RA
IL-1RA (pg/ml)
IL-6
IL-6 (pg/ml)
IL-8
IL-8 (pg/ml)
I-TAC
I-TAC (pg/ml)
MCP-1
MCP-1 (pg/ml)
MIP-3a
MIP-3a (pg/ml)
sFas
sFas (pg/ml)
TARC
TARC (pg/ml)
TRAIL
TRAIL (pg/ml)
VEGF-A
VEGF-A (pg/ml)
6 h
24 h
Time of IAV addition
IAV
OC43
OC43+IAV

Figure S9

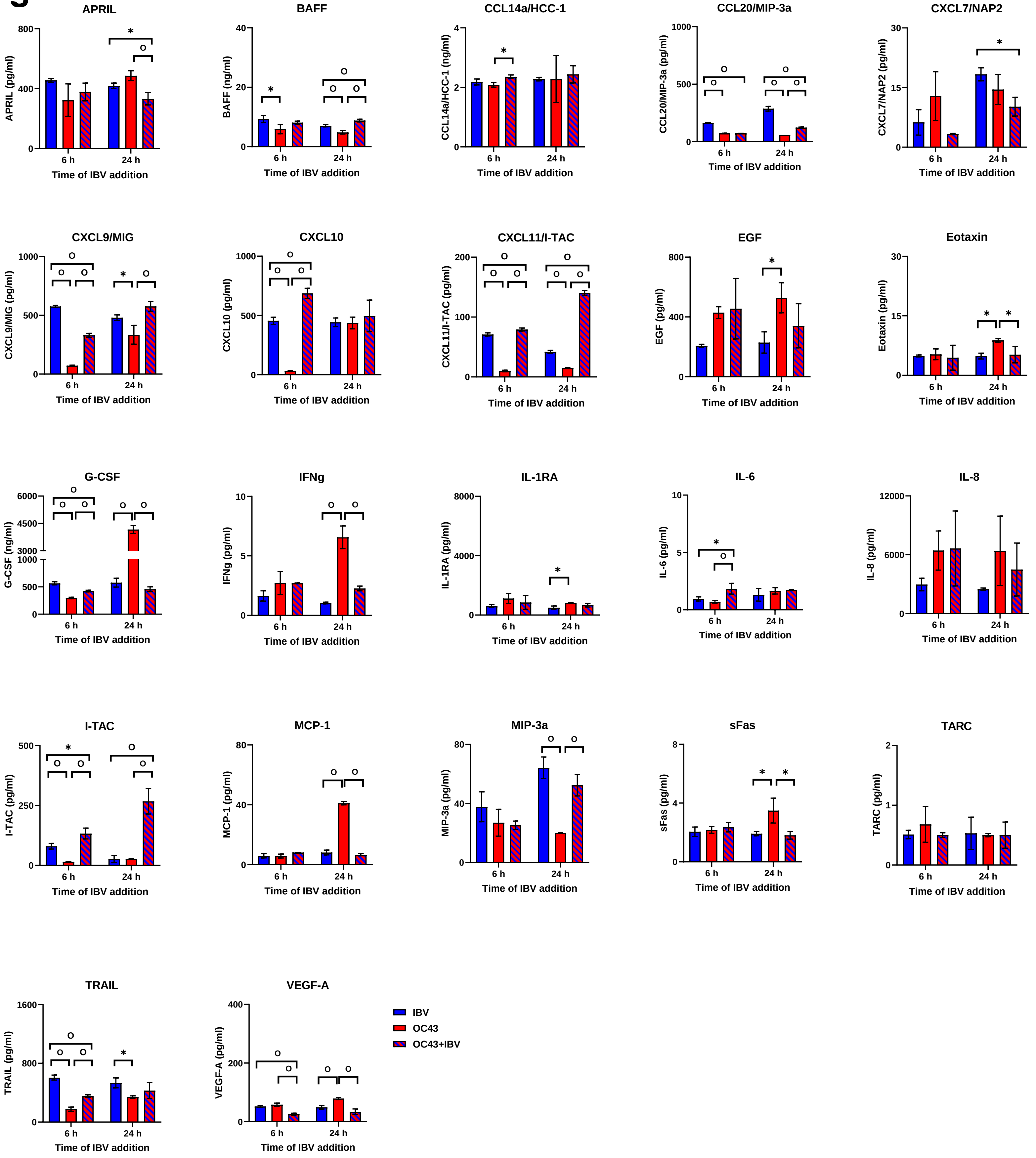

APRIL
APRIL (pg/ml)
BAFF
BAFF (ng/ml)
CCL14a/HCC-1
CCL14a/HCC-1 (ng/ml)
CCL20/MIP-3a
CCL20/MIP-3a (pg/ml)
CXCL7/NAP2
CXCL7/NAP2 (pg/ml)
CXCL9/MIG
CXCL9/MIG (pg/ml)
CXCL10
CXCL10 (pg/ml)
CXCL11/I-TAC
CXCL11/I-TAC (pg/ml)
EGF
EGF (pg/ml)
Eotaxin
Eotaxin (pg/ml)
G-CSF
G-CSF (ng/ml)
IFNg
IFNg (pg/ml)
IL-1RA
IL-1RA (pg/ml)
IL-6
IL-6 (pg/ml)
IL-8
IL-8 (pg/ml)
I-TAC
I-TAC (pg/ml)
MCP-1
MCP-1 (pg/ml)
MIP-3a
MIP-3a (pg/ml)
sFas
sFas (pg/ml)
TARC
TARC (pg/ml)
TRAIL
TRAIL (pg/ml)
VEGF-A
VEGF-A (pg/ml)
6 h
24 h
Time of IBV addition
IBV
OC43
OC43+IBV
